# Prediction of SYNTAX score II improvement by adding temporal heart rate changes between discharge and first outpatient visit in patients with acute myocardial infarction

**DOI:** 10.1186/s12872-022-02929-7

**Published:** 2022-11-08

**Authors:** Chuang Li, Wanjing Zhang, Yixing Yang, Qian Zhang, Kuibao Li, Mulei Chen, Lefeng Wang, Kun Xia

**Affiliations:** Heart Center and Beijing Key Laboratory of Hypertension, Beijing Chaoyang Hospital, Capital Medical University, 8# Gong-Ti South Road, Beijing, 10020 China

**Keywords:** Temporal changes in resting heart rate, Major adverse cardiovascular events, Acute myocardial infarction, SYNTAX score

## Abstract

**Background:**

The prognostic ability of the temporal changes in resting heart rate (ΔHR) in patients with acute myocardial infarction (AMI) for cardiovascular (CV) mortality and clinical outcomes is rarely examined. This study investigated the predictive value of ΔHR using models with SYNTAX score II (SxS-II) for the long-term prognosis of patients with AMI.

**Methods:**

Six hundred five AMI patients with vital signs recorded at the first outpatient visit (2–4 weeks after discharge) were retrospectively recruited into this study. The changes between discharge and outpatient resting heart rate (D-O ΔHR) were calculated by subtracting the HR at the first post-discharge visit from the value recorded at discharge. The major adverse cardiovascular and cerebrovascular events (MACCE) include cardiovascular death, recurrent myocardial infarction, revascularization, and nonfatal stroke. The predictive values and reclassification ability of the different models were assessed using a likelihood ratio test, Akaike’s information criteria (AIC), receiver operating characteristic (ROC) curves, net reclassification improvement (NRI), and integrated discrimination improvement (IDI).

**Results:**

During the follow-up period, a drop-in resting heart rate (RHR) from discharge to first outpatient visit was independently associated with less risk of CV mortality [D-O ΔHR: hazards ratio (HR) = 0.97, 95% CI = 0.96–0.99, *P* < 0.001] and MACCE (HR = 0.98, 95% CI = 0.97–0.99, *p* = 0.001). The likelihood test indicated that the combined model of SxS-II and D-O ΔHR yielded the lowest AIC for CV mortality and MACCE (*P* < 0.001). Moreover, D-O ΔHR alone significantly improved the net reclassification and integrated discrimination of the models containing SxS-II for CV mortality and MACCE (CV mortality: NRI = 0.5600, *P* = 0.001 and IDI = 0.0759, *P* = 0.03; MACCE: NRI = 0.2231, *P* < 0.05 and IDI = 0.0107, *P* < 0.05).

**Conclusions:**

The change in D-O ΔHR was an independent predictor of long-term CV mortality and MACCE. The D-O ΔHR combined with SxS-II could significantly improve its predictive probability.

## Introduction

An elevated resting heart rate (RHR) is a risk factor for cardiovascular (CV) morbidity and mortality in the general population. It is associated with various clinical scenarios, such as chronic heart failure (HF), myocardial infarction (MI), cancer, etc. [1–4]. RHR reduction is associated with clinical benefits after myocardial infarction [5], regardless of using beta-blockers and calcium channel blockers. Recently, a series of large-scale trials indicated that the short-term temporal changes in resting HR (ΔHR) within three months or less after myocardial infarction showed a better correlation with CV mortality and adverse outcomes of HF [2, 6, 7]. However, limited evidence was documented for the magnitude of association between ΔHR recorded at discharge and that measured at the first outpatient visit and the long-term prognosis of patients with acute myocardial infarction (AMI).

Additionally, the Synergy between the percutaneous coronary intervention (PCI) with Taxus and Cardiac Surgery (SYNTAX) score II (SxS-II) has been recommended for the identification of patients with a high CV risk among a population with multivessel or left main coronary artery disease (CAD) and for aiding in decision making for different coronary revascularization strategies [8, 9]. Some studies on the predictive possibility of SxS-II on clinical CV mortality and other events related to AMI were documented [10–14]. However, the SxS-II risk system lacks consideration of the instantaneous clinical variables, such as the changes in RHR, which is vital in the risk stratification of the AMI [15, 16]. Therefore, this study assessed the predictive performance of the ΔHR recorded between discharge and the first outpatient visit for the prognosis of AMI patients who underwent PCI and to explore the prognostic value of a combined model including ΔHR and the SxS-II system to reclassify the risk of further CV events.

## Methods

### Patient selection

Of 6592 patients who underwent PCI with a confirmed diagnosis of AMI, 635 consecutive cases with their vital signs recorded at the first post-discharge visit (2–4 weeks) at the Beijing Chaoyang Hospital Heart Center from January 2014 to June 2019 were retrospectively recruited. The diagnostic criteria of AMI were done according to the fourth universal definition of myocardial infarction [17]: (1) a significant elevated cardiac troponin I (cTnI) at least the 99^th^ percentile upper reference limit; (2) typical ischemic symptoms; and (3) a newly developed left bundle branch block pattern or a new ST-segment elevation or depression in two or more contiguous leads, with readings of at least 0.2 mV in leads V1, V2, and V3 or of at least 0.1 mV in the remaining leads. We excluded the patients with arrhythmia, atrial fibrillation, pacemakers, a life expectancy of less than six months, a history of coronary artery bypass (CABG), dialysis, or cirrhosis.

### Outpatient vital sign collection and SYNTAX score II calculation

The RHR was recorded in parallel with the blood pressure measurement using a blood pressure monitor (OMRON HBP-9020 or HEM-7137, Omron, Shandong, China). The HRs measured at admission and discharge were presented as a mean of two RHR values recorded by a physician. The temporal changes in RHR from admission and discharge resting heart rate (A-D ΔHR) were calculated by subtracting the HR at discharge from the HR at admission. During their follow-ups after discharge (2–4 weeks), outpatient vital signs were measured twice at a 1-min interval after the participant had rested for 5 min with the arm resting on a table. We calculated temporal changes in resting HR (D-O and A-O ΔHR) by subtracting the HR recorded at the first post-discharge visit (oHR) from the value recorded at admission or discharge (shown in Table 1). All other baseline information, including clinical features, demographics, and treatment records, were collected from the medical database of the Beijing Chaoyang Hospital.

**Table 1:** Definitions of heat rates changes at a different time point in recruited patients with acute myocardial infarction

Term	Abbreviation	Measurement
RHR at admission	Admission HR	It was recorded in parallel to the measurement of blood pressure after resting for 5 min at admission. The mean value of two RHR values measured with a 1-min interval was presented
RHR at discharge	Discharge HR	It was measured after lying for 5 min in the morning at discharge and recorded as a mean of two RHR values measured with a 1- min interval
RHR at first outpatient visit after AMI	Outpatient HR	It was measured after resting for 5 min at the first outpatient visit 2–4 weeks following AMI and recorded as a mean of two RHR values with a 1-min interval
Changes in RHR between admission and discharge	A-D ΔHR	A-D ΔHR = RHR at admission–RHR at discharge
Changes in RHR between admission and first outpatient visit	A-OΔHR	A-O ΔHR = RHR at admission–RHR at first outpatients visit
Changes in RHR between discharge and first outpatient visit	D-O ΔHR	D-O ΔHR = RHR at discharge–RHR at first outpatients visit

*RHR* resting heart rate, *AMI* acute myocardial infarction

In this study, coronary angiography was retrospectively reviewed by two cardiologists. They were blinded to the outcomes and management of the patients during the follow-up. The SYNTAX score was calculated using the initial angiogram based on the SxS calculator. All lesions of stenosis ≥ 50% in the main branch or major side branch (≥ 1.5 mm in diameter) were scored. Patients with a history of coronary artery bypass grafting were excluded. Subsequently, SxS-II was calculated based on two anatomical variables (SxS and left the main CAD) and six clinical variables [age, gender, chronic obstructive pulmonary disease (COPD), peripheral arterial disease, creatinine clearance, and left ventricular ejection fraction (LVEF)] using an automatic online calculation system.

### Follow-up and clinical endpoint

All subjects were followed-up via telephone contact, scheduled outpatient visits, or the medical records after the first outpatient visit. The primary endpoint was defined as MACCE, which mainly comprised CV death, recurrent nonfatal MI, repeat coronary revascularization, and nonfatal ischemic stroke. CV mortality was defined as death with cardiac problems. The recurrent nonfatal MI was defined as the recurrence of ischemic symptoms or new ECG changes of ST-segment elevation or depression ≥ 0.1 mV in at least two continuous leads with the elevated cardia biomarker ≥ 20% preceding values. Repeated revascularization was defined as revascularization driven by clinical ischemia symptoms. The staged PCI was excluded from revascularization.

### Statistical analysis

The continuous variables were presented as the mean with a standard deviation (mean ± SD) or median with interquartile range (IQR), and categorical variables were presented as the frequency with percentage. Student’s *t*-test was conducted to compare the variables with a Gaussian distribution. The Mann–Whitney *U* or Kruskal–Wallis nonparametric tests were used to compare the non-normally distributed variables. The Chi-squared test was used to detect differences in categorical variables. The Kaplan–Meier survival curves were implemented to evaluate the incidence of the clinical endpoint, whereas the log-rank test was used to detect intergroup differences. Cox proportional hazard regression analyses were used to identify predictors of CV mortality and MACCE. The variance inflation factor (VIF) was calculated for each independent variable. High collinearity was considered when VIF > 10.

ROC curves were generated to assess the predictive value of SxS-II and combined models. Due to the limited ability of classical ROC curve analysis, the time-dependent ROC curves were performed in this study [18]. Moreover, the NRI and IDI were used to evaluate the degree to which the different types of ΔHR improved the predictive ability of the SxS-II model [19]. The event NRI (NRIe) was defined as the net percentage of patients with an event that was correctly assigned a higher predicted risk, whereas the nonevent NRI (NRIne) was defined as the net percentage of patients without an event that was correctly assigned a lower predicted risk. The likelihood ratio test was used to assess whether the combined models could provide a better prognostic value. AIC was measured to identify the “best fitting” combined model [20]. Statistical analyses were performed using STATA (version 15.0) and the R statistical software (version 3.4.0). All statistical tests were two-tailed, and a *P*-value ≤ 0.05 was considered statistically significant.

## Results

### Baseline clinical characteristics and incidence of MACCE

After screening the outpatient documents and angiogram, we excluded four cases with incomplete data, ten cases with a history of CABG, and six cases with atrial fibrillation or pacemaker implantation. Finally, 605 AMI patients with detailed HR records at the first post-discharge visit met the inclusion criteria. Subsequently, all recruited patients were categorized into two groups according to the median of heart rate changes between discharge and the first post-discharge visit [D-O ΔHR ≤  − 1 beat per minute (bpm) group and ΔHR >  − 1 bpm group]. Patients in the D-O ΔHR ≤  − 1 bpm group were older, had a higher proportion of females, presented with lower diastolic blood pressure (DBP) at admission and discharge, a lower RHR at discharge and outpatient, higher counts of white blood cells, and increased level of high sensitivity C-reactive protein (hs-CRP) (Table 2). Moreover, their presentation was complicated by a higher prevalence of hypertension and diabetes mellitus and lower proportions of β-blocker and statin therapy. No significant differences in the baseline characteristic were observed between the two ΔHR groups.

**Table 2:** Baseline characteristic according to the median of temporal change values in heart rate between discharge and first outpatient visit

Factor	Total	D-O ΔHR ≤ − 1 (beats/min)	D-O ΔHR > − 1 (beats/min)	*p*-value
N	605	314	291	
Age (year, IQR)	62 (54, 71)	63 (54, 72)	62 (51, 69)	0.06
Male, n (%)	463 (76.5)	229 (72.9)	234 (80.4)	0.04
BMI, (kg/m^2^, SD)	25.4 (3.68)	25.4 (3.26)	25.4 (4.09)	0.79
Diagnosis, n (%)				0.25
STEMI	592 (97.9)	308 (98.1)	284 (97.6)	
NSTEMI	13 (2.1)	6 (1.8)	7 (2.4)	
Killip, n (%)				0.64
I	283 (46.8)	145 (46.2)	138 (47.4)	
II	262 (43.3)	140 (44.6)	122 (41.9)	
III	33 (5.5)	18 (5.7)	15 (5.2)	
IV	27 (4.5)	11 (3.5)	16 (5.5)	
LVEF, %	61 (52, 67)	61 (51, 66)	61 (52, 67)	0.29
Outpatient SBP, (mmHg)	126 (16.6)	127 (17.6)	125 (15.4)	0.07
Outpatient DBP, (mmHg)	74 (9.8)	74 (11.3)	73 (8.0)	0.96
Admission SBP, (mmHg)	124 (20.4)	123 (18.2)	125 (22.5)	0.27
Admission DBP, (mmHg)	71 (12.7)	70 (11.8)	73 (13.4)	0.03
Discharge SBP, (mmHg)	122 (13.5)	122 (13.0)	122 (14.1)	0.70
Discharge DBP, (mmHg)	71 (9.0)	70 (8.5)	72 (9.5)	0.04
Admission HR, (beat/min)	76 (67, 85)	76 (68, 86)	75 (65, 84)	0.21
Discharge HR, (beat/min)	70 (65, 76)	66 (63, 70)	72 (69, 79)	< 0.001
Outpatient HR, (beat/min)	70 (65, 77)	75 (70, 80)	66 (60, 70)	< 0.001
A-D ΔHR, (beat/min)	− 1 (1, 20)	7 (− 1, 16)	− 2 (− 7, 6)	< 0.001
A-O ΔHR, (beat/min)	4 (− 5, 14)	− 1 (− 10, 9)	9 (1, 18)	< 0.001
**Medical history**				
Prior MI, n (%)	70 (11.6)	33 (10.5)	37 (12.7)	0.40
Diabetes mellitus, n (%)	199 (32.9)	119 (37.9)	80 (27.5)	< 0.01
Hypertension, n (%)	337 (55.7)	191 (60.8)	146 (50.2)	< 0.01
Hyperlipoidemia, n (%)	150 (24.8)	74 (23.6)	76 (26.1)	0.47
COPD, n (%)	52 (8.6)	26 (8.3)	26 (8.9)	0.77
PVD, n (%)	4 (0.7)	4 (1.3)	0 (0)	0.12
Smoker, n (%)	358 (59.2)	188 (59.9)	170 (58.4)	0.72
**Laboratory test**				
White blood cell, (10*9/L)	10.8 (2.86)	11.1 (2.89)	10.6 (2.80)	0.02
Precent of neutral cell, (%)	79.8 (10.07)	79.9 (9.86)	79.7 (10.3)	0.79
Hemoglobin, (10*9/L)	134 (16.9)	135 (15.9)	133 (18.0)	0.23
Platelet count, (10*9/L)	216 (57.3)	219 (57.2)	212 (57.1)	0.11
Cholesterol, (mmol/L)	4.58(1.08)	4.62 (1.03)	4.53 (1.12)	0.11
HDL, (mmol/L)	1.1 (0.29)	1.1 (0.26)	1.1 (0.32)	0.96
LDL, (mmol/L)	2.88 (0.89)	2.91 (0.89)	2.84 (0.88)	0.34
Triglyceride, (mmol/L)	1.32 (0.95, 1.86)	1.34 (0.92, 1.78)	1.3 (0.96, 1.92)	0.72
Fasting glucose, (mmol/L)	6.7 (5.43, 9.01)	6.63 (5.4, 9.06)	6.77 (5.52, 8.96)	0.95
BNP, (pg/ml)	195 (58.6, 642)	245 (64, 656)	145 (55.3, 638)	0.10
CTNI, (ng/ml)	31.28(9.79, 76.6)	30.4 (10.07, 79.85)	31.99 (9.61, 88.21)	0.65
Hs-CRP, (mg/dl)	4.5 (2.14, 10.99)	5.3 (2.26, 11.3)	3.8 (2.0, 10.6)	0.02
Creatinine, (mmol/L)	74.1(64.4, 87.4)	74.5 (65.6, 87.4)	74.1 (64.3, 87.1)	0.71
**Medication at discharge**				
Aspirin, n (%)	604 (99.8)	314 (100.0)	290 (99.7)	0.30
Clopidogrel, n (%)	587 (97.0)	306 (97.5)	281 (96.6)	0.52
Ticagrelor, n (%)	18 (3.0)	8 (2.5)	10 (3.4)	0.95
β-blocker, n (%)	380 (62.9)	180 (57.5)	200 (68.7)	< 0.01
ACEI/ARB, n (%)	310 (51.2)	158 (50.3)	152 (52.2)	0.64
Statin, n (%)	545 (90.1)	272 (86.6)	273 (93.8)	< 0.01
Nitrogen, n (%)	179 (29.6)	95 (30.3)	84 (28.9)	0.71
SYNTAX score	26.32 (9.85)	26.69 (9.68)	25.92 (10.04)	0.34
SYNTAX score II	27.6 (23, 37)	28 (23, 38)	27.6 (22.8, 36)	0.16

*Abbreviation*: *BMI* body mass index, *STEMI* ST-segment elevated myocardial infraction, *NSTEM* non-ST-segment elevated myocardial infraction, *LVEF* left ventricular ejection fraction, *HR* heart rate, *SBP* systolic blood pressure, *DBP* diastolic blood pressure, *MI* myocardial infarction, *PCI* percutaneous coronary intervention, *BNP* brain natriuretic peptide, *CTNI* cardiac troponin I, *HDL* High-density lipoprotein, *LDL* Low-density lipoprotein, *Hs-CRP* high sensitivity C-reactive protein, *ACEI* angiotensin-converting enzyme inhibitors, *ARB* angiotensin receptor blocker, *SYNTAX* Synergy between PCI with Taxus and Cardiac Surgery

**Fig. 1:**
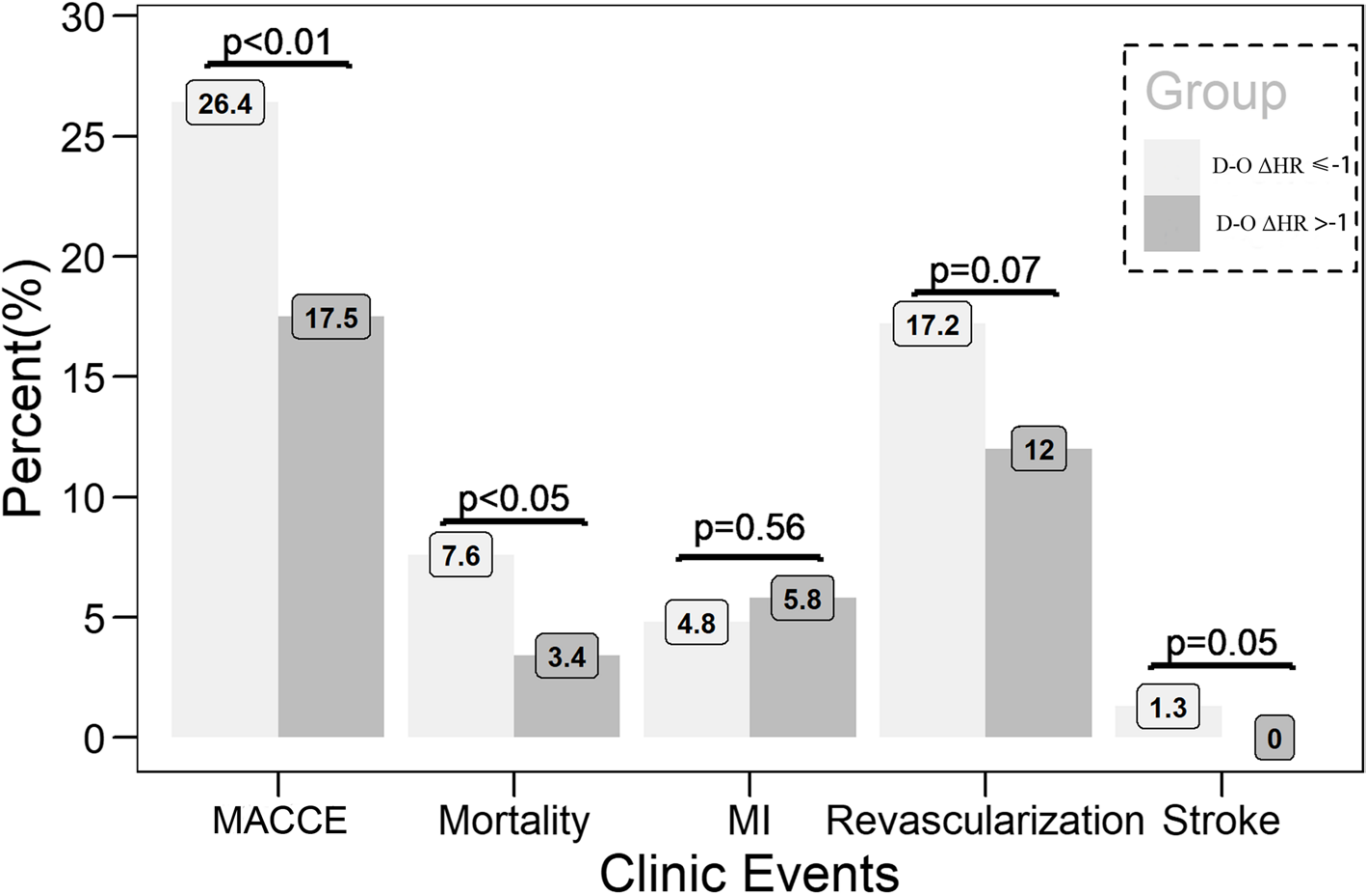
Comparison of clinical endpoints among the two D-O ΔHR groups. Patients with the D-O ΔHR ≤ -1 bpm had a higher incidence of MACCE, CV mortality, and nonfatal stroke

During the median follow-up period of 26 months (IQR: 16–38 months), 134 (22.1%) cases exhibited adverse clinical events, including 34 (5.6%) CV deaths, 32 (5.3%) recurrent MIs, 89 (14.7%) repeat revascularizations, and 4 (0.7%) nonfatal strokes. The overall MACCE and CV death incidences were significantly higher in the D-O ΔHR ≤  − 1 bpm group than D-O >  − 1 bpm group (26.4% *vs.* 17.5%, *P* < 0.01; 7.6% *vs.* 3.4%, *P* < 0.05, respectively). Patients in the D-O ΔHR ≤  − 1 bpm group were prone to complications, as they exhibited a higher frequency of repeat revascularizations (17.2% *vs.* 12.0%, *P* = 0.07) and ischemic stroke (1.3% *vs.* 0%, *P* = 0.05) (Fig. 1).

### Kaplan–Meier survival curves and Cox regression analysis for MACCE and CV mortality

**Fig. 2:**
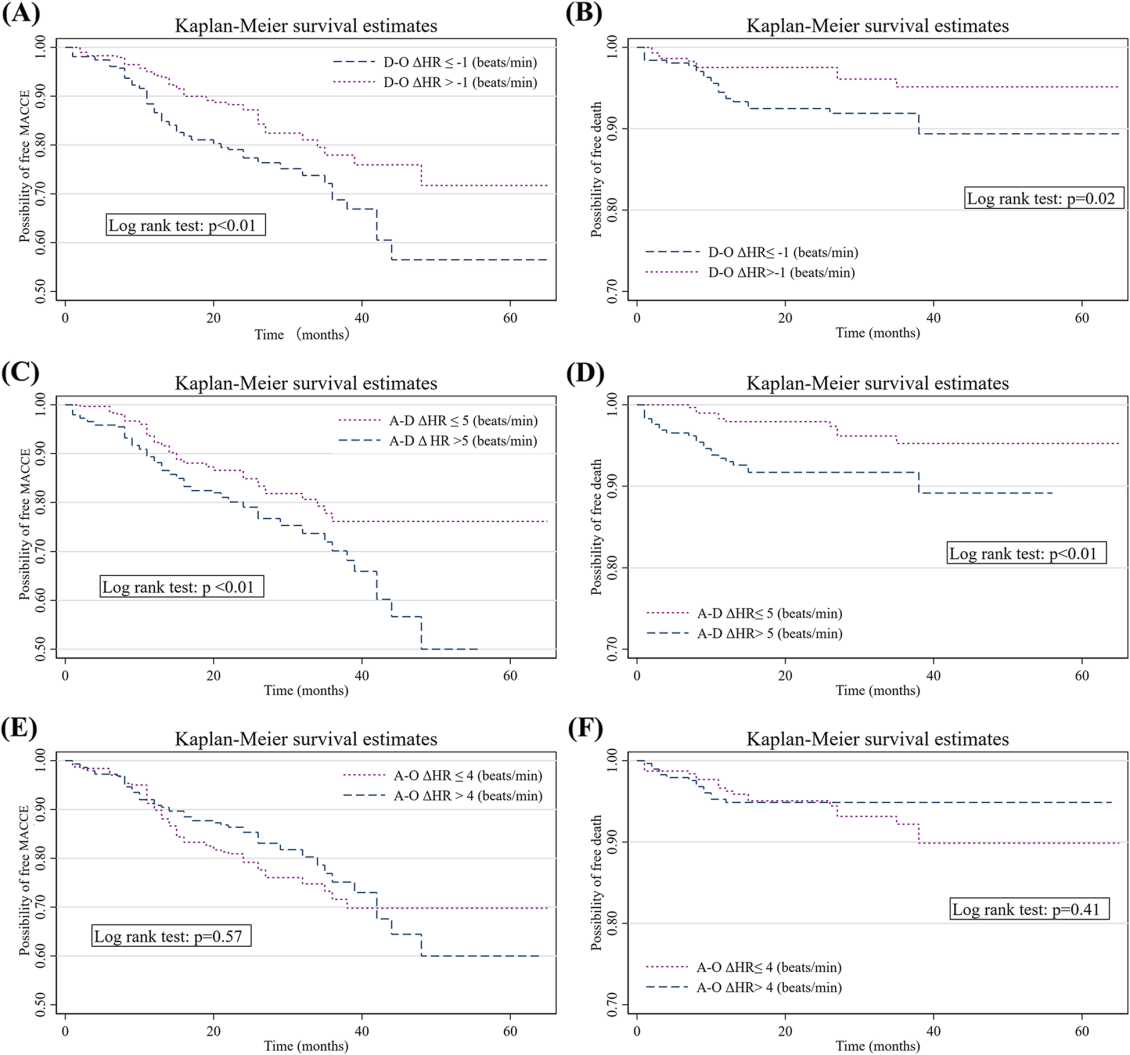
Kaplan–Meier failure curve of MACCE and CV mortality during the median follow-up period of 26 months according to D-O, A-O, and A-D ΔHR, respectively. Patients with the D-O ΔHR > -1 bpm were favorable to free MACCE (**A**) and CV mortality (**B**). Patients with A-D ΔHR > 5 bpm were prone to suffer MACCE (**C**) and CV mortality (**D**). No significant discrepancy among two group divided by the median of A-O ΔHR was observed on MACCE (**E**) and CV mortality (**F**)

Kaplan–Meier survival analysis was used to assess the cumulative survival for MACCE and CV mortality in the two groups, which were divided according to the median D-O ΔHR and A-D ΔHR (Fig. 2). During the observational period, patients with D-O ΔHR >  − 1 bpm had a significantly lower incidence of MACCE and CV deaths than patients with D-O ΔHR ≤  − 1 bpm group (log-rank test: *P* < 0.01 and *P* = 0.02, respectively) (Fig. 2A and B). Moreover, a similar phenomenon was observed in the two groups by dividing by the median A-D ΔHR (log-rank test: *P* < 0.01 for MACCE and CV deaths) (Fig. 2C and D). In contrast, the cumulative survival rates for MACCE and CV deaths were not significantly different between the two groups that were divided by the median A–O ΔHR (Fig. 2E and F).

The results of the Cox proportional hazard regression analysis are shown in Table 3. The univariate analysis revealed that RHR at the outpatient visit (HR = 1.02, 95% CI = 1.01–1.03, *P* < 0.001; HR = 1.03, 95% CI = 1.02–1.04, *P* < 0.001) and admission (HR = 1.01, 95% CI = 1.00–1.02, *P* = 0.02; HR = 1.02, 95% CI = 1.00–1.04, *P* = 0.03), and A–D ΔHR (HR = 1.01, 95% CI = 1.00–1.02, *P* = 0.02; HR = 1.02, 95% CI = 1.00–1.04, *P* = 0.02) were associated with an increased risk of MACCE and CV death, respectively. The D–O ΔHR was a protector against MACCE and CV death (HR = 0.98, 95% CI = 0.97–0.99, *P* < 0.001; HR = 0.96, 95% CI = 0.95–0.98, *P* < 0.001, respectively), whereas a high A–O ΔHR was exclusively associated with a decrease in CV death (HR = 0.97, 95% CI = 0.96–0.99, *P* = 0.001). No multicollinearity was observed between the different types of ΔHR and its continuous parameters (i.e., SxS, age, LVEF, and creatinine) based on the VIF. The multivariate analysis after adjustment for other potential confounders, including sex, age, creatine level, ejection fraction, SYNTAX score, HGB level, use of beta-blocker, history of diabetes mellitus, hypertension, hyperlipidemia, PCI, and prior MI, indicated that the D–O ΔHR (HR = 0.97, 95% CI = 0.96–0.99, *P* < 0.01), A–O ΔHR (HR = 0.98, 95% CI = 0.97–1.00, *P* = 0.05), and RHR at the first outpatient visit (HR = 1.02, 95% CI = 1.01–1.04, *P* < 0.01) remained independent powerful predictors of the CV morality. Regarding the incidence of MACCE, the D–O ΔHR (HR = 0.98, 95% CI = 0.97–0.99, *P* < 0.01), A–D ΔHR (HR = 1.01, 95% CI = 1.00–1.02, *P* = 0.05), and RHR at the first outpatient visit (HR = 1.01, 95% CI = 1.01–1.02, *P* < 0.01) remained as an independent predictor of outcomes in patients with AMI.

**Table 3:** Cox proportional hazard regression analyses for major adverse cardiovascular events and CV mortality

**Model**^a^	**Univariate analysis**		**Multivariable analysis**	
**MACCE**	**HR (95% CI)**	***P*-value**	**HR (95% CI)**	***P*-value**
Outpatient HR	1.02 (1.01—1.03)	< 0.001	1.01 (1.01—1.02)	< 0.01
Admission HR	1.01 (1.00—1.02)	0.02	1.01 (0.99—1.02)	0.08
Discharge HR	0.99 (0.97—1.02)	0.63	0.99 (0.97—1.02)	0.53
A-D ΔHR	1.01 (1.00—1.02)	0.02	1.01 (1.00—1.02)	0.05
A-O ΔHR	0.99 (0.98—1.00)	0.25	0.99 (0.99—1.00)	0.36
D-O ΔHR	0.98 (0.97—0.99)	< 0.001	0.98 (0.97—0.99)	< 0.01
**Mortality**				
Outpatient HR	1.03 (1.02—1.04)	< 0.001	1.02 (1.01—1.04)	< 0.01
Admission HR	1. 02 (1.00—1.04)	0.03	1.01 (0.99—1.04)	0.19
Discharge HR	0.98 (0.94—1.03)	0.42	0.97 (0.92—1.02)	0.31
A-D ΔHR	1.02 (1.00—1.04)	0.02	1.02 (0.99—1.04)	0.09
A-O ΔHR	0.97 (0.96—0.99)	0.001	0.98 (0.97—1.00)	0.05
D-O ΔHR	0.96 (0.95—0.98)	< 0.001	0. 97 (0.96—0.99)	< 0.01

*Abbreviations*: *HGB* hemoglobin, *PCI* percutaneous coronary intervention, *MI* myocardial infarction

^a^Model: Adjustment of the confounding factors, including sex, age, creatine level, ejection fraction, SYNTAX score, HGB level, use of beta-blocker, history of diabetes mellitus, hypertension, hyperlipidemia, PCI and prior MI

### Combination of SxS-II with different types of ΔHR values for predicting clinical outcomes

Based on the likelihood ratio test, models of SxS-II and D–O ΔHR exhibited the best fit with the lowest AIC compared with the remaining four models for MACCE (AIC: 1553.823, *P* < 0.001) and CV death (AIC: 390.9558, *P* < 0.001) (Table 4).

**Table 4:** Akaike’s information criteria and likelihood ratio to determine the best fitting model for predicting MACCE and cardiovascular death

		**AIC**	**Likelihood ratio test**		
**Clinical outcomes**	Model	AIC	χ^2^	df	*P*-value
**MACCE**	SxS- II	1561.39			
	SxS-II + oHR	1555.927	7.46	1	< 0.01
	SxS-II + D-OΔHR	1553.823	9.57	1	< 0.01
	SxS-II + A-DΔHR	1557.27	4.29	1	0.04
	SxS-II + A-OΔHR	1562.754	0.64	1	0.43
**CV death**	SxS-II	404.5431			
	SxS-II + oHR	394.4685	12.07	1	< 0.001
	SxS-II + D-OΔHR	390.9558	15.59	1	< 0.001
	SxS-II + A-DΔHR	403.2508	2.96	1	0.09
	SxS-II + A-O ΔHR	400.9642	5.58	1	0.02

*Abbreviations*: *AIC* Akaike’s information criteria, *SxS- II* SYNTAX score II, *MACCE* major adverse cardiovascular events

**Fig. 3:**
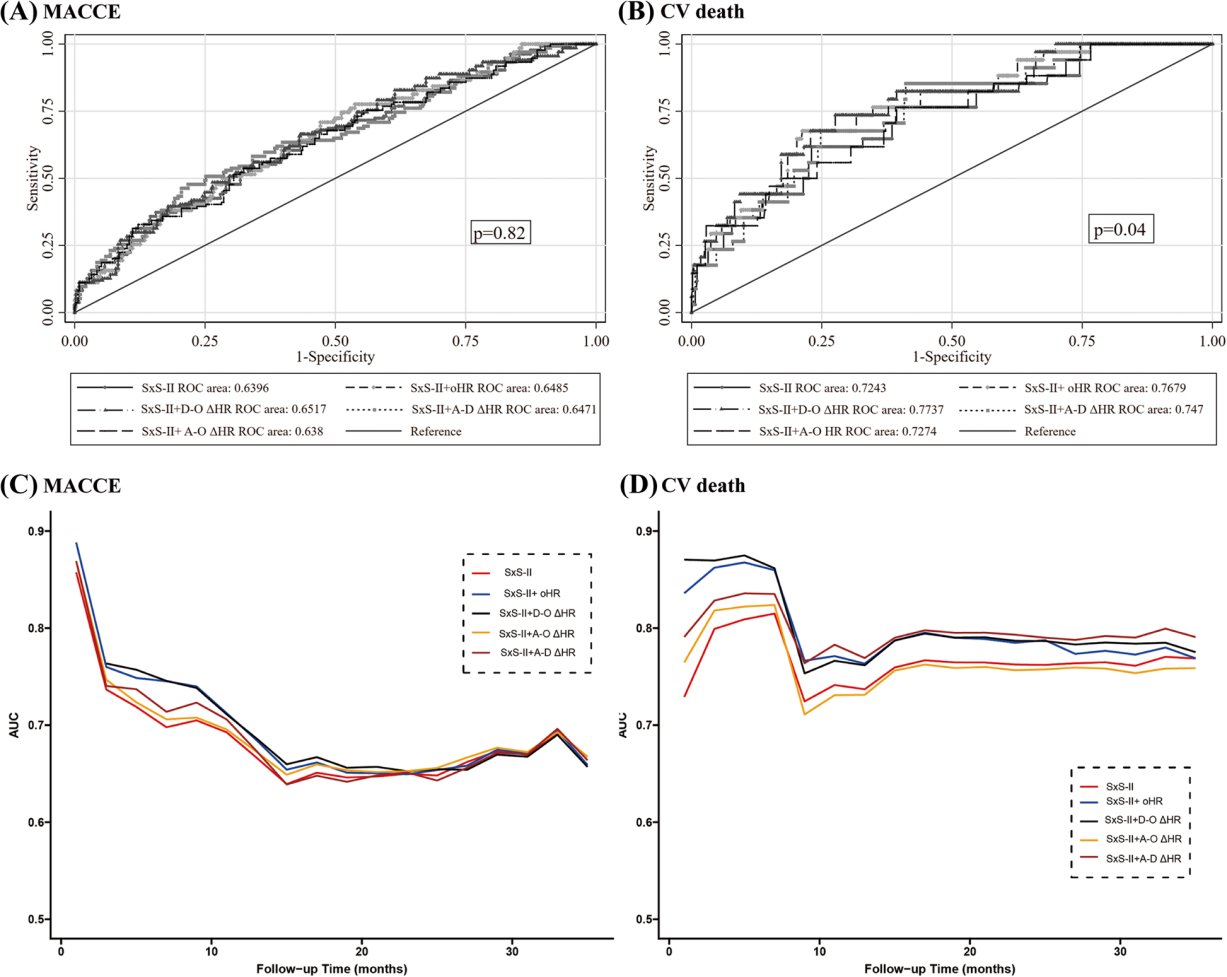
Conventional and time-dependent receiver operating characteristic (ROC) curve for the combined models and SYNTAX score II (SxS-II) alone in predicting long-term MACCE (**A**, **C**) and CV mortality (**B**, **D**), respectively. Combined models containing SxS-II and D-O ΔHR provided the best predictive value significantly

The incremental prognostic value of the incorporation of the different types of ΔHR values into SxS-II for MACCE and CV mortality was measured based on the increase in the area under the ROC curve (AUC) (Fig. 3A and B). Regarding MACCE, no significant difference was observed among the AUC of the models (SxS-II: 0.6396 *vs.* SxS-II + oHR: 0.6485 *vs.* SxS-II + D–O ΔHR: 0.6517 *vs.* SxS-II + A–D ΔHR: 0.6471 *vs.* SxS-II + A–O ΔHR: 0.638; *P* = 0.82). Except for the SxS-II and A–O ΔHR models, the AUC of the combination of the three remaining models for CV death was 0.7679, 0.7737, and 0.747, respectively, of which all were grossly higher than that recorded for SxS-II alone (*P* = 0.04). According to the tendency analyzed by the time-dependent ROC curve, the AUC containing SxS-II and D–O ΔHR could provide a durable and competent predictive ability for MACCE and CV deaths (Fig. 3C and D).

The results obtained from NRI and IDI indicated that the models of SxS-II and D–O ΔHR exhibited a significantly better net reclassification improvement in predicting MACCE and CV mortality (Table 5). Compared with the other combined models, models of SxS-II and D–O ΔHR provided a significant improvement of 2.99% in the reclassification of patients with MACCE and an improvement of 19.32% in the classification of those without MACCE (*P* < 0.05). In total, the NRI of the models of SxS-II and D–O ΔHR were 22.31% and 56.0% for MACCE and CV deaths, respectively. Regarding CV death, the two combined models of SxS-II and D–O ΔHR or oHR resulted in a significant discrimination and reclassification improvement in the nonevent and event groups (17.64% and 38.36%, and 29.42% and 46.76%, respectively; *P* < 0.001). However, the IDI analysis indicated that the predictive value for MACCE and CV death was only significantly improved by the incorporation of D–O ΔHR into the SxS-II model (MACCE: 0.0107, *P* < 0.05; CV death: 0.0759, *P* = 0.03).

**Table 5:** Net reclassification improvement for model improvement with the addition of different types of heart rate discrepancy to SxS-II

**MACCE**						
Models	NRIe	NRIne	NRI total	*P*-value	IDI total	*P*-value
SxS-II + oHR	− 0.2239	0.3546	0.1307	0.18	0.0096	0.09
SxS-II + D-OΔHR	0.0299	0.1932	0.2231	< 0.05	0.0107	< 0.05
SxS-II + A-OΔHR	0.00	0.0064	0.0064	0.95	0.0008	0.25
SxS-II + A-DΔHR	0.0149	0.096	0.1109	0.25	0.0058	0.10
**Cardiovascular mortality**						
Model	NRIe	NRIne	NRI total	*P*-value	IDI total	*P*-value
SxS-II + oHR	0.2942	0.4676	0.7617	< 0.001	0.0627	0.07
SxS-II + D-OΔHR	0.1764	0.3836	0.5600	0.001	0.0759	0.03
SxS-II + A-OΔHR	− 0.2352	0.1874	− 0.0478	0.61	0.0323	0.15
SxS-II + A-DΔHR	0.1764	0.1704	0.3469	0.05	0.0077	0.08

*Abbreviations*: *NRI* net reclassification index, *IDI* integrated discrimination improvement, *SxS-II* SYNTAX score II, *HR* heart rate, *MACCE* major adverse cardiovascular and cerebrovascular events

## Discussion

Our findings identified the association between three ΔHRs recorded at a different time points during the acute phase of MI and long-term adverse clinical events in patients with AMI. After adjustment for other relevant confounding factors, the temporal RHR changes recorded between discharge and the first outpatient visit (2–4 weeks) presented an independent predictor of MACCE and CV mortality. Moreover, models combining D–O ΔHR and the SxS-II have improved the predictive value for long-term CV death and MACCE. Additionally, the calibration, discriminatory capacity, and reclassification of the SxS-II model were significantly updated by the integration of D–O ΔHR.

The association between changes in RHR and CV death and other outcomes was previously assessed in populations without a known CV disease [3, 4, 21]. In the community-based cohort study reported by Vazir [2], individuals with an increase in ΔHR within a median interval of 3 years exhibited a greater risk of CV mortality and other clinical outcomes. Moreover, findings from the Treatment of Preserved Cardiac Function Heart Failure with an Aldosterone Antagonist Study (TOPCAT) [7] confirmed that increased RHR over a median of 3 months was associated with a higher risk of CV deaths, hospitalization for HF, and stroke in patients with HF with a preserved ejection fraction, with the exception of recurrent MI. Thereby, many recent studies have demonstrated that the predictive value of changes in RHR for CV mortality and other clinical events is similar to or better than that of RHR [1, 4, 22]. However, few studies have investigated the predictive value of the magnitude of ΔHR for CV outcomes in post-myocardial infarction. This was the first study to describe an association between long-term CV deaths and other events, and changes in RHR from discharge to outpatients visit in AMI patients who underwent PCI. Moreover, the result was compatible with the assumption that D–O ΔHR at the early stage of AMI is likely to be a predictor of CV outcomes.

Our observations suggest the prognostic value of RHR changes between discharge and the first outpatient visit, independently of beta-blocker treatment. In this study, majority of the patients were preserved LVEF and received a high frequency of beta-blocker therapy. Most recently, Joo’s study found no association between beta-blocker treatment and outcomes on post-MI with regard to preserved LVEF among enrolled 13,624 patients [23]. In essence, the cardiac output stimulated by an increased RHR was elevated within 7 days and, followed by decling within 1 to 2 months after AMI in a rat model [24]. Moreover, further beneficial effects of RHR reduction on the left ventricular diastolic pressure and increased ejection fraction were observed within 1 to 2 months. The magnitude of RHR reduction during the period from discharge to 2–4 week outpatients visit is associated with reduced myocardial oxygen consumption, adequate coronary and myocardial perfusion, and rehabilitation of impaired cardiac function and subendocardial blood flow [1, 25, 26] and proportionally related to the clinical benefit of beta-blocker or calcium channel blockers on post-infarction [5]. Hence, the magnitude of change in RHR during the acute phase of AMI is an efficient and convenient parameter to predict the worse prognosis and should be paid attention, independently of beta-blocker treatment.

The SxS-II score comprised coronary anatomy and clinical risk factors. Therefore, it was better than the SYNTAX score for decision making on the treatment of left main CAD or complex three-vessel disease according to the European Society of Cardiology (ESC) guidelines [8, 27]. Recent studies showed that the predictive ability of the SxS-II for long-term CV mortality is excellent in patients with STEMI and is superior to other risk-scoring systems, such as the GRACE and TIMI scores [10, 12, 28, 29]. Moreover, all the variables involved in SxS-II could not reflect the elevated sympathetic tone triggered by the deterioration of HF or a cascade of inflammatory reactions that contributed to the worsened prognosis of AMI. Given the intimate association of the increased RHR with long-term MACCE, CV mortality, and HF in patients with AMI [30–32], we hypothesized that the model complemented the changes in RHR between different time points with the SxS-II improves the accuracy of risk classification. Therefore, the D–O ΔHR grossly provided a more significant improvement in risk stratification and reclassification of clinical outcomes, which was better than the RHR at the post-discharge visit. Although a more significant improvement in net reclassification was found after adding the RHR at the first outpatient visit to the SxS-II system, the IDI analysis devised by Pencina et al*.* [33] for evaluating reclassification indicated that these matrixes improved when the D–O ΔHR is added to the SxS-II. In conclusion, our analysis provided a comprehensive and logical rationale for determining the utility of the temporal changes in HR as a biomarker for optimizing the predictive ability of the SxS-II.

## Limitations

Some of the limitations were present in this study. First, because of the retrospective nature, a selection bias was inevitable and affected our findings. Particularly, the patients who died in the acute phase or were severely disabled were excluded from the analysis. Moreover, the high proportion of STEMI patients in this study may cause selection bias. Therefore, a large prospective study with a subgroup analysis must address these issues in the future. Second, previous studies indicated that the RHR at discharge is associated with CV mortality and clinical outcomes; however, no similar phenomenon was observed in our study. This may be attributed to the controlled RHR at discharge, with a median of 70 beats/min, which yields a similar risk of CV mortality to that of an RHR of 60 beats/min [31]. Hence, we speculate that the discrepancy between the two RHR values recorded at different time points may increase the predictive value. Finally, as RHR or ΔHR is associated with various chronic inflammation disorders, such as asthma, inflammatory bowel disease, and vasculitis, it is uncertain whether ΔHR is potentially influenced by the inflammation status. However, in the context of the hyperactivity of inflammation disorders, the RHR could still evaluate the severity of inflammation activity and predict coronary atherosclerosis and cardiac mortality [34].

## Conclusions

Our evidence suggests that D–O ΔHR was a robust independent predictor of adverse clinical outcomes in AMI patients who underwent PCI after adjusting for conventional CV risk factors. Moreover, incorporating the ΔHR recorded between discharge and the first outpatient visit into the SxS-II system improves the predictive ability of patients with AMI after PCI for long-term CV mortality and MACCE.

## Data Availability

The datasets generated and analyzed during this study are not publicly available due to the policies of Beijing Chaoyang Hospital on individual confidentiality but are available from the corresponding author upon reasonable request.
